# Risk factor analysis for early femoral failure in metal-on-metal hip resurfacing arthroplasty: the effect of bone density and body mass index

**DOI:** 10.1186/1749-799X-7-1

**Published:** 2012-01-10

**Authors:** Thomas P Gross, Fei Liu

**Affiliations:** 1 Midlands Orthopaedics, p.a. Columbia, SC, USA

## Abstract

**Background:**

The importance of appropriately selecting patients based on factors such as bone mineral density, body mass index, age, gender, and femoral component size has been demonstrated in many studies as an aid in decreasing the rate of revisions and improving the outcomes for patients after hip resurfacing arthroplasty (HRA); however, there are few published studies quantitatively specifying the potential risk factors that affect early femoral component failures. Therefore, the purpose of this study was to investigate the specific causes of early femoral component failures in hip resurfacing separately and more carefully in order to develop strategies to prevent these failures, rather than excluding groups of patients from this surgical procedure.

**Methods:**

This retrospective study included 373 metal-on-metal HRAs performed by a single surgeon using the vascular sparing posterior minimally invasive surgical approach. The average length of follow-up was 30 ± 6 months. In order to understand the causes of early femoral failure rate, a multivariable logistic regression model was generated in order to analyze the effects of bone mineral density (T-score), gender, diagnosis, body mass index, femoral implant fixation type, age, and femoral component size.

**Results:**

The average post-operative Harris hip score was 92 ± 11 points and the average post-operative UCLA score was 7 ± 2 points. There were three revisions due to femoral neck fracture and two for femoral component loosening. These occurred in two female and three male patients. In the multi-variable regression model, only T-score and body mass index showed significant effects on the failure rate of femoral components. Patients with a lower T-score and a higher body mass index had a significantly increased risk of early femoral component failure.

**Conclusion:**

We recommend that dual energy x-ray absorptiometry scan T-score tests should be routinely performed on all hip resurfacing patients pre-operatively. If a patient has a low T-score (≤ -1.5), consideration should be given to additional precautions or treatments to alleviate his or her risk, especially when the patient has a higher body mass index (≥ 29 kg/m^2^).

## Background

Metal-on-metal hip resurfacing arthroplasty (HRA) has become an established alternative to traditional total hip arthroplasty (THA) for younger, more active patients within the last decade. Recently, as many at 10% of hip arthroplasties worldwide were reported to be performed using resurfacing implants [1,2]. Clinical studies have demonstrated successful early to midterm results (1-10 years) with survivorship rates ranging from 93.2% to 99.8% [3-6]. The proposed advantages of this procedure are enhanced stability due to the implementation of larger anatomic bearing size and increased preservation of the femoral neck, which may also make femoral revision comparable to primary femoral replacement in THA [3].

Some studies have showed increased complication rates with this procedure, especially for inexperienced surgeons [2,7]. While a number of studies have suggested an association between various patient and implant characteristics and an increased rate of failure, few have specifically quantified independent risk factors. Furthermore, risk factors have been most commonly studied with respect to all failure modes of hip resurfacing [3,8,9]. The most common modes of failures are early femoral component failures occurring before two years after hip resurfacing. This includes femoral neck fractures and femoral component loosening, which is suspected to take place as a result of the following thermal osteonecrosis of the underlying bone [2,10]. Both of these complications are unique to hip resurfacing procedures and neither occurred in stemmed THAs. It is most likely that the risk factors that apply to early femoral component failure are different than those that apply to other modes of failure, such as acetabular loosening and adverse wear. Although the importance of appropriately selecting patients based on factors such as bone mineral density, body mass index, age, gender, and femoral component size has been demonstrated in many studies as an aid in decreasing the rates of revision and improving patient outcome, there are few published studies quantitatively specifying which risk factors independently affect early failure of femoral components. Therefore, we wanted to investigate the specific causes of early femoral component failures in hip resurfacing separately and more carefully in order to develop strategies to prevent them rather than excluding groups of patients from HRA.

Based on a single surgeon's experience with metal-on-metal HRA, the purpose of this study was: (1) to report our clinical results of a group of consecutive metal-on-metal HRA cases for which bone mineral density was recorded and alendronate was not administered; (2) to identify the underlying causes associated with an increased early femoral failure after hip resurfacing by using multivariable logistic regression models; and (3) to use univariate analysis to determine thresholds for each risk factor to make them clinically useful as well as analyze the combined effects of these factors in order to predict failure rates by using reduced model analysis based on the determined thresholds.

## Methods

Before this study, the senior author (T.P.G.) performed 830 HRAs since 1999 [11]. Therefore, by most published criteria, he had already surpassed the learning curve of hip resurfacing procedures prior to this study. Beginning in July 2006, we routinely recorded bone mineral density with the use of a dual energy x-ray absorptiometry (DEXA) scan prior to metal-on-metal HRAs. After September 2008, we started treating low bone density patients with alendronate. In this retrospective study, 373 consecutive metal-on-metal HRAs were implanted in 346 patients by the senior author between July 2006 and September 2008. Bone mineral density data (T-score) were recorded for all of these cases, and none of the cases were treated with alendronate. Two patients (two hips) died from unrelated causes. Because their two-year follow-up information was available, they were still included in the study. 233 (67%) patients were men, the average age was 52 ± 8 years (range: 23 to 76 years), the average body mass index was 27 ± 4 kg/m^2 ^(range: 18 to 43 kg/m^2^), and the average DEXA scan T-score was 0.09 ± 1.4 (range from -2.4 to 6.7). The primary diagnosis was osteoarthritis in 290 hips (78%) followed by dysplasia in 52 hips (14%), osteonecrosis in 14 cases (4%), post-traumatic arthritis in 8 cases (2%), Legg-Calve-Perthes in three cases (0.8%), slipped capital femoral epiphysis in three cases (0.8%), post-infection in one case (0.3%), rheumatoid arthritis (RA) in one case (0.3%), and ankylosing spondylitis in one case (0.3%). Pre-operative demographic information, Harris hip scores, and intra-operative technical data were routinely collected in this study. Follow-up visits were requested at six weeks, one year, two years, and every other year thereafter post-operatively. The average length of follow-up in the present study was 30 ± 6 months (range: 24 to 47 months). Post-operative information including post-operative Harris hip scores, visual analog scale (VAS) pain scores on regular days and on worst days, UCLA activity scores, complications, and failures were prospectively recorded for all patients. Anteroposterior and lateral radiographs were also routinely analyzed at each follow-up visit. All of the above data were maintained in a computerized database, OrthoTrack (Midlands Orthopaedics, p.a., Columbia, SC). Institutional review board approval (IRB) was obtained for this study.

The senior surgeon used a previously described [12] posterior, minimally invasive surgical approach on all cases. In 77% of these cases, a Biomet ReCap™ cemented femoral component (Biomet, Warsaw, IN, USA) was used while in the remaining 23%, a ReCap™ fully porous coated femoral component was used. The average femoral component size was 50 ± 4 mm (range: 40 to 60 mm). Fully porous coated Magnum™ acetabular components were used in all cases, and their outer diameter sizes were 6 mm larger than the corresponding femoral component. The average acetabular inclination angle was 42° ± 7° (range: 19° to 61°).

A paired *t*-test was utilized to compare the statistical difference between the pre- and post- operative HHS score. Kaplan-Meier survivorship curves [13] were calculated using femoral failure, acetabular failure, or both for any reason as the end points, respectively, in order to analyze the success rates of up to four-year follow-up in this study. However, the primary endpoint studied was any femoral failure that occurred before two years post-operatively. This included all femoral neck fractures and all less acute femoral failures that were evident clinically or radiographically before two years. If a patient was revised or had radiographic signs of femoral failure at up to three years post-operatively, they were included as an early failure if their symptoms or radiographic abnormalities were present prior to two years post-operatively. All of the following statistical analyses used only early femoral failure for any reason as the end point. Multivariable logistic regression models were generated to identify significant risk factors for early femoral failure after metal-on-metal HRA. In this logistic regression model, early femoral failure was a categorical variable and defined as the outcome. Age, gender, diagnosis, body mass index, T-score, femoral implant fixation type, and the size of the femoral component were each defined as explanatory variables. These explanatory variables of age, gender, body mass index, T-score, and the size of the femoral component were initially included as categorical variables grouped with different thresholds according to our experience or suggested by previous references [8,9,14,15], as well as numerical variables. Different multivariable logistic regression models were tested by changing the types and thresholds of these variables in order to find the best regression model to predict the early femoral failures. The final regression model determined whether these five variables should be treated as category variables and, if so, what the thresholds should be. First, a full factorial regression model including all seven factors was generated to help us predict the possibility of early femoral failure. Covariates, which did not contribute significantly to the model fit with the significance level α = 0.05, were excluded from the present model. Then, a reduced regression model was built to evaluate which independent factor had the strongest effect on the failures. Possibilities for femoral failures within the ranges of these independent risk factors were predicted based on this reduced model and plotted to determine their effects. Finally, the significant risk factors were regrouped with different thresholds. Mosaic plots were depicted and Chi-square analyses were performed to evaluate the thresholds of each risk factor and thecombined factors in order to provide more meaningful information for surgeons for clinical use.

## Results

**Figure 1:**
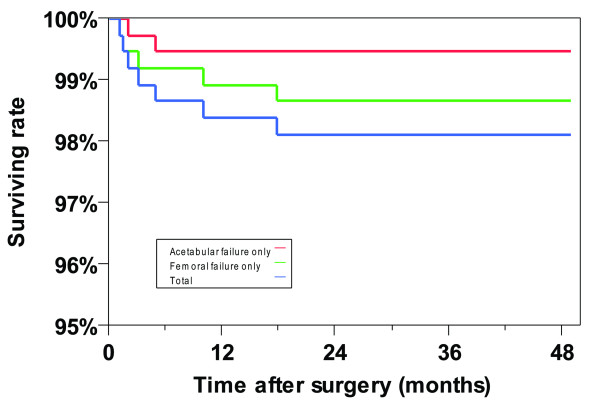
**Kaplan-Meier survivorship curves with use of femoral component failure, acetabular component failure, or either for any reason taken as the end point**.

The Harris hip scores for patients significantly improved after surgery (pre-operative: 55 ± 14 points vs. post-operative: 92 ± 11 points; P > 0.001) with great pain relief (visual analog scale pain score: 0 ± 1 points on the regular day and 1 ± 2 points on the worst day) and high activity levels (UCLA activity score: 7 ± 2 points). In total, there were seven failures (1.9%). Five (1.4%) femoral failures were identified. There were three femoral neck fractures and two femoral component loosenings prior to two years after surgery (Table 1). All of these cases were treated with femoral revisions to THA with retention of the acetabular component. The four-year cumulative survivorship rate was 98.1%, 99.5%, and 98.6% with use of femoral component failure, acetabular component failure, or either for any reason taken as the end point, respectively (Figure 1). In addition to the failures, there were seven cases that experienced hip-related complications that did not require revision: one deep infection (0.3%), two shifted acetabular components (0.6%), three hip dislocations (0.8%), and one abductor tear four months post-operatively with minimal trauma (0.3%). There was no radiolucency or osteolysis observed on the femoral side in the remaining cases. Two cases (0.5%) were revised due to acetabular component loosening: one was in a male patient with a T-score of 2.3 and a body mass index of 28 kg/m^2^, which was revised to a THA at two months post-operatively; the other case was in a female patient with a T-score of -2.1 and body mass index of 28 kg/m^2^, for which only the acetabular component was revised at 4.9 months post-operatively. There was no radiolucency or osteolysis observed on the acetabular side in the remaining cases.

**Table 1:** Detailed information of early femoral component failures.

Time after surgery (Months)	Reason of Failure	Femoral Fixation	T Score	Femoral Size (mm)	Primary Diagnosis	Sex	Body mass index (kg/m^2^)	Age (yrs)
1	Femoral Neck Fracture	Fully porous coated	-1.9	52	OA	Male	35	59

1.4	Femoral Neck Fracture	Fully porous coated	-0.3	44	OA	Female	24	61

3.1	Femoral Neck Fracture	Cemented	-1.6	46	OA	Female	29	43

10	Femoral Loosening	Fully porous coated	-0.5	54	Dysplasia	Male	34	50

17.8*	Femoral Loosening	Cemented	-2.1	52	AVN	Male	31	31

* The primary indication of hip resurfacing was avascular necrosis for this young gentleman. The symptom of severe pain due to avascular necrosis into femoral component was found 17.8 months post-operatively with visual analog scale pain score of 9/10. The patient waited to revise to total hip arthroplasty 27.6 months after surgery.

In our final multivariable logistic regression models, age, body mass index, and the size of the femoral component were treated as numerical variables; T-score was treated as a categorical variable and grouped into three categories: T ≥ 0, 0 > T > -1.5, and T ≤ -1.5; diagnosis was treated as a categorical variable and was divided into the two groups of Osteoarthritis and Not Osteoarthritis; femoral implant fixation type was included as a categorical variable and divided into the groups of Cemented or Uncemented. In our full seven-factor multivariable regression model (*P *> Chi-sq = 0.04; lack of fit *P *> Chi-sq = 1.0), only T-score (*P *= 0.002) and body mass index (*P *= 0.04) showed significant effects on the failure rate of femoral components (Table 2). Age, gender, implant size, diagnosis, and femoral fixation type (implant type) did not contribute significantly to the prediction of an early femoral failure in our regression model. After removing these factors, the reduced two-factor regression model (*P *> Chi-sq = 0.002; lack of fit *P *> Chi-sq = 1.0), which only included T-score and body mass index, fit as well as the above mentioned seven-factor full regression model, demonstrating that T-score had the strongest effect on predicting the failure of femoral components (*P *= 0.002) and that the body mass index had a significant effect on it (*P *= 0.02). According to the full and reduced regression model, a lower T-score and a greater body mass index increase the risk of an early femoral component failure.

**Table 2:** Summary of the full and reduced multivariable logistic regression model.

Variables	Degree of Freedom	Type*	*P *value
**Full model including all of the following variables (P = 0.04)**			

Femoral fixation type	1	C	0.7

Femoral Component Size	1	N	0.5

Primary DX	1	C	0.95

Sex	1	C	0.27

Age	1	N	0.67

T-score	2**	C	**0.002**

BMI	1	N	**0.02**

**Reduced model only including significant variables (P = 0.002)**			

T-score	2**	C	**0.002**

BMI	1	N	**0.02**

* C = Category; N = Numerical

** Grouped with T≥0, 0 > T > -1.5, and T≤ -1.5

**Figure 2:**
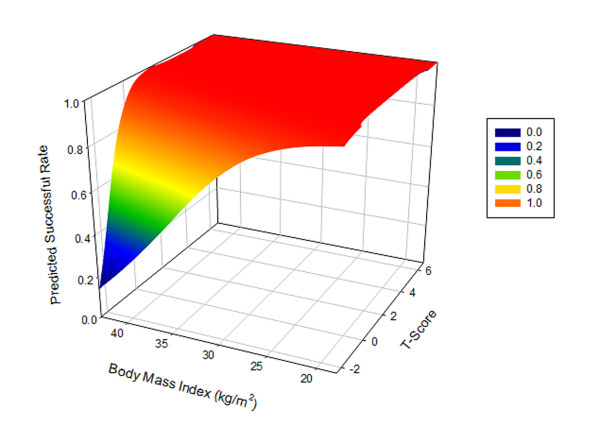
**Correlation of the success rate as a function of T-score and BMI**.

Univariate analysis demonstrated that T-score = -1.5 and body mass index = 29 were the thresholds that affect early femoral component failures (Table 3). The correlation between the predicted failure based on our multivariable logistic regression model and the explanatory variables of T-score and body mass index demonstrated that the femoral failure rate could be as high as 87% if a patient has a T-score of -2.4 and a body mass index of 43 kg/m^2 ^(Figure 2).

**Table 3:** Risk analysis between failure rates with T-score, body mass index, or combined.

Variable	Threshold	Failure Rate	Percentage	*P*-Value
T-score	T ≥ 0	0/172	0%	0.003
		
	0 > T > -1.5	2/161	1.2%	
		
	T ≤ -1.5	3/40	8%	

Body mass index (kg/m^2^)	< 29	1/234	0.4%	0.05
		
	≥ 29	4/139	2.9%	

Combined	T < -1.5 & BMI ≥ 29	3/12	25%	< 0.001
		
	Others	2/361	0.6%	

## Discussion

The most commonly reported complication in hip resurfacing, particularly in the first two years, is early femoral failure [7,16,17]. This takes form as acute fracture or gradual collapse of the femoral head within the first two years. Normally, studies combine all types of failures when analyzing the risk factors for HRA. It is not only possible, but also likely, that the causes that underlie other modes of failure are different than those that are causative for early femoral failure. The strength of this retrospective study is that only one failure mode is analyzed in this prospectively collected database where numerous risk factors have been recorded. In the present study, the combined early femoral failure rate was 1.4% (5/373) at an average three-year follow-up. By selectively analyzing only one failure mode, we can get a more accurate idea of the underlying causes of this specific complication. In the present study, DEXA T-score is the factor most predictive of early femoral failure after hip resurfacing. The other factor that was found to be predictive of failure was increased body mass index. Other factors previously linked to a higher failure rate in hip resurfacing, such as increased age, female gender, and smaller component size were not found to be independent predictors of early femoral failure in our study. Whether or not the femoral component was fixed by means of cement or bone ingrowth did not affect the failure rate. Based on the present study, patients with a low T-score (≤ -1.5) [risk rate = 7.9%, relative risk = 6.3 times higher] or a high BMI (≥29) [risk rate = 2.9%, relative risk = 7.3 times higher] should be considered at higher risks for the complication of early femoral failure after HRA (Table 3). When these risk factors are combined, the risk is particularly high [risk rate = 25%, relative risk = 42 times higher].

The following weaknesses of our study were recognized. Firstly, all of the cases in this study were done through a vascular sparing posterior approach. Numerous studies have suggested that partial devascularization of the proximal femur during surgery may lead to proximal femoral failures. Some surgical approaches have been suggested to be more vascular sparing to the proximal femur. However, no comparative studies have demonstrated that one approach is less likely to cause proximal femoral failure. Since we are not able to analyze the influence of a surgical approach on early femoral failure, the findings from this study may not apply to other commonly used approaches. Secondly, the primary diagnosis was osteoarthritis (77%). There were only twelve cases with the primary diagnosis of osteonecrosis and 48 with dysplasia. We suspect that patients with these two primary diagnoses may have a higher risk for early femoral component failures [18]. The significance, however, could not be drawn from this study possibly due to the lack of sufficient patient population. Even one national registry did not find a difference based on diagnosis [19]. This may suggest that although some diagnoses may be predisposed to early femoral failure, the effect appears to be weak, requiring large numbers of patients to demonstrate failures with such diagnoses. Thirdly, a single experienced hip resurfacing surgeon performed all of the cases. The causes of early femoral failure when the learning curve has not been completed may include other risk factors. Even so, DEXA scans are an established method of measuring bone density. The T-score relates bone density to young, healthy, race and gender-matched bone. It is easily obtained and provides an objective quantitative number. The T-score and body mass index are not influenced by an observer (surgeon) bias. Therefore, findings from this study can easily be incorporated into the practice of hip resurfacing without mastering additional skills. Finally, only five early femoral component failures occurred in this study. However, lower T-score and higher BMI were identified as the significant risk factors even in this small series. According to the statistical analysis, further large series should be performed in order to confirm the outcome of this study; but, caution has been taken immediately by surgeons who are performing these proceduresfor patients with lower T-score combined with higher BMI in order to reduce the early failure rate of femoral components.

No study has previously provided scientific evidence that low bone density is related to early femoral failure. Despite this lack of direct evidence, patients with reduced bone density are generally considered to have a higher risk for femoral neck fracture. No threshold has been previously suggested [3,20]. To the best of our knowledge, this is the first time a commonly used measure of bone strength has been quantitatively analyzed to assess whether it can predict early femoral failure. Based on the unilateral statistical analysis, 0 and -1.5 were suggested as the thresholds to predict the early femoral component failures. None of the patients with T-score ≥0 had early femoral failure in this study. Significantly more patients had early femoral failures with T-scores ≤-1.5. This confirms that patients with weaker bone are more likely to suffer from the most common early complications of hip resurfacing. Evidence has been given that the femoral bone mineral density decreased significantly by three months after metal-on-metal Birmingham HRAs ((Smith&Nephew, Memphis, TN, USA)); thereafter, it stopped decreasing and began increasing six months post-operatively [21,22]. This is not surprising in light of what we know about the biology of fracture healing. As supported by these data, it seems logical to believe that increased bone mineral density prevents patients from femoral neck fractures six months after hip resurfacing surgeries. Also, bone mineral density (*P *= 0.002) showed a stronger effect in our regression model on the early femoral neck fracture compared to body mass index (*P *= 0.02), which suggests that bone mineral density was the more critical factor associated with femoral neck fracture when compared to body mass index. This also suggests that high levels of activity should be discouraged until at least six months after surgery, when it is known that bone density returns to normal. Comparatively, the two acetabular component failures occurred in one patient with a high T-score (2.3), and the other with a low T-score (-2.1), which may not suggest that T-score affects the survivorship of acetabular components as significantly as femoral components after hip resurfacing.

At the same time, the risk increases when the patient is overweight and places added stress on the weakened bone. Although Amstutz's studies suggested that lower weight or lower body mass index increases failure rates [15,23], our results demonstrated the opposite opinion and was consistent with others [14,24]. It is logical, though no evidence has been presented, that a body mass index ≥ 35 kg/m^2 ^increases the complexity of exposing the hip and accurately placing the component, therefore increasing the risk in femoral notching [3,14]. Our study indicated that a higher body mass index (> 29 kg/m^2^) significantly increased the chance of a femoral failure.

## Conclusion

Our study suggests that low patient bone mineral density could be one of the primary causative factors for early femoral failure after hip resurfacing. Greater body mass index could be the other underlying cause that increases the risk of this complication. We recommend obtaining a pre-operative DEXA scan of the operative femoral neck and calculating the body mass index on every patient who is considering hip resurfacing. Patients should be counseled regarding their risk of femoral failure based on the T-score and body mass index values (Table 3). Frequently, patient selection is practiced to avoid hip resurfacing in patients determined to have a higher risk of complications with this operation. However, caution must also be used with this approach because it has not been determined that patients with lower bone density do not also have a higher risk of periprosthetic fracture after stemmed THA. An alternative approach that should be investigated is to modify treatment in this high-risk group in order to mitigate their risk.

## Competing interests

The authors declare that they have no competing interests.

## Authors' contributions

TPG designed this study, collected the data, and drafted the manuscript. FL designed this study, analyzed the data, performed statistical analyses and drafted the manuscript. All of the authors read and approved the final version of this study.
